# Supplementary material for: A transcriptomic taxonomy of mouse brain-wide spinal projecting neurons
Source: Nature. 2023 Dec 13;624(7991):403–14. doi: 10.1038/s41586-023-06817-8 (PMC10719099; doi:10.1038/s41586-023-06817-8)
Supplement: Supplementary file 2 — Reporting Summary [file 41586_2023_6817_MOESM2_ESM.pdf]

nature portfolio

Corresponding author(s): Zhigang He, Hongkui Zeng, Anne Jacobi

Last updated by author(s): Nov 20, 2023

Reporting Summary

Nature Portfolio wishes to improve the reproducibility of the work that we publish. This form provides structure for consistency and transparency in reporting. For further information on Nature Portfolio policies, see our Editorial Policies and the Editorial Policy Checklist.

Statistics

For all statistical analyses, confirm that the following items are present in the figure legend, table legend, main text, or Methods section.

n/a	Confirmed	
☐	☒	The exact sample size (*n*) for each experimental group/condition, given as a discrete number and unit of measurement
☐	☒	A statement on whether measurements were taken from distinct samples or whether the same sample was measured repeatedly
☐	☒	The statistical test(s) used AND whether they are one- or two-sided<br>*Only common tests should be described solely by name; describe more complex techniques in the Methods section.*
☐	☒	A description of all covariates tested
☐	☒	A description of any assumptions or corrections, such as tests of normality and adjustment for multiple comparisons
☐	☒	A full description of the statistical parameters including central tendency (e.g. means) or other basic estimates (e.g. regression coefficient) AND variation (e.g. standard deviation) or associated estimates of uncertainty (e.g. confidence intervals)
☐	☒	For null hypothesis testing, the test statistic (e.g. *F*, *t*, *r*) with confidence intervals, effect sizes, degrees of freedom and *P* value noted<br>*Give P values as exact values whenever suitable.*
☒	☐	For Bayesian analysis, information on the choice of priors and Markov chain Monte Carlo settings
☒	☐	For hierarchical and complex designs, identification of the appropriate level for tests and full reporting of outcomes
☒	☐	Estimates of effect sizes (e.g. Cohen's *d*, Pearson's *r*), indicating how they were calculated

Our web collection on statistics for biologists contains articles on many of the points above.

Software and code

Policy information about availability of computer code

Data collection	Software for mapping and analysis of transcriptomic datasets is fully described in the Methods.<br><br>Confocal imaging data was acquired with Zen 3.3 and LAS X Stellaris. Slide scanner imaging data was acquired with VS-ASW. Serial two-photon tomography data was acquired with TissueVision's imaging software. Imaging data was analyzed using QuPath version 0.4.1 and NeuroInfo version 2023-1-1.<br><br>Electrophysiology data was recorded with pClamp software (Version 11, Molecular Devices).<br><br>Code availability: Code to reproduce analyses here is available at https://github.com/ZhigangHeLab/SPN_atlas. Additional code used in the manuscript is available at https://github.com/AllenInstitute/scrattch.bigcat and https://github.com/AllenInstitute/scrattch.mapping.
Data analysis	Software for mapping and analysis of transcriptomic datasets is fully described in the Methods.<br>Software for analysis of imaging datasets (confocal, slide-scanner, and serial two-photon tomography) is fully described in the Methods.

For manuscripts utilizing custom algorithms or software that are central to the research but not yet described in published literature, software must be made available to editors and reviewers. We strongly encourage code deposition in a community repository (e.g. GitHub). See the Nature Portfolio guidelines for submitting code & software for further information.

Data

Policy information about availability of data

All manuscripts must include a data availability statement. This statement should provide the following information, where applicable:

- Accession codes, unique identifiers, or web links for publicly available datasets
- A description of any restrictions on data availability
- For clinical datasets or third party data, please ensure that the statement adheres to our policy

The data are accessible through the Neuroscience Multi-omics (NeMO) Archive (https://assets.nemoarchive.org/dat-76h044v) and the Gene Expression Omnibus (GEO; accession number GSE247602). The AIBS WB atlas data are accessible through NeMO (https://assets.nemoarchive.org/dat-qg7n1b0).

Research involving human participants, their data, or biological material

Policy information about studies with human participants or human data. See also policy information about sex, gender (identity/presentation), and sexual orientation and race, ethnicity and racism.

Reporting on sex and gender	This study did not involve human participants.
Reporting on race, ethnicity, or other socially relevant groupings	This study did not involve human participants.
Population characteristics	This study did not involve human participants.
Recruitment	This study did not involve human participants.
Ethics oversight	This study did not involve human participants.

Note that full information on the approval of the study protocol must also be provided in the manuscript.

Field-specific reporting

Please select the one below that is the best fit for your research. If you are not sure, read the appropriate sections before making your selection.

☒ Life sciences ☐ Behavioural & social sciences ☐ Ecological, evolutionary & environmental sciences

For a reference copy of the document with all sections, see nature.com/documents/nr-reporting-summary-flat.pdf

Life sciences study design

All studies must disclose on these points even when the disclosure is negative.

Sample size	Sample size (number of animals) was determined by the experimental requirements for collection of sufficient tissue for each assay. For snRNA-seq data, nuclei suspensions were generated from 15 adult mice (8 female and 7 male) for 10x and 15 mice (6 female and 9 male) for SSv4. We did not observe differences between individual animals or batches. The number of nuclei collected was determined by limitations of each data modality.<br><br>For electrophysiology and imaging experiments, no statistical methods were used to predetermine sample size.
Data exclusions	For transcriptomic data, low quality nuclei were excluded based on criteria that are detailed in the Methods. Briefly, nuclei were filtered to retain only those with less than 5% mitochondrial counts and more than 2000 genes detected. These are standard thresholds used in previous studies.
Replication	No data point from electrophysiology data was excluded.<br><br>Findings from SSv4 and 10x platforms were compared across biological replicates. We did not observed any disagreement between replicates.
Randomization	Randomization is not applicable for our study since it does not involve a comparison between treatment and control groups. This study characterizes SPN cell types in untreated mice. snRNA-seq provides a random selection of nuclei from the original tissue source.
Blinding	Blinding was not needed as there were no treatment and control groups.

Reporting for specific materials, systems and methods

We require information from authors about some types of materials, experimental systems and methods used in many studies. Here, indicate whether each material, system or method listed is relevant to your study. If you are not sure if a list item applies to your research, read the appropriate section before selecting a response.

Materials & experimental systems

n/a	Involved in the study
☐	☒ Antibodies
☒	☐ Eukaryotic cell lines
☒	☐ Palaeontology and archaeology
☐	☒ Animals and other organisms
☒	☐ Clinical data
☒	☐ Dual use research of concern
☒	☐ Plants

Methods

n/a	Involved in the study
☒	☐ ChIP-seq
☐	☒ Flow cytometry
☒	☐ MRI-based neuroimaging

Antibodies

Antibodies used

Anti-Green Fluorescent Protein Antibody, AVES, SKU: GFP-1020
RFP Antibody Pre-adsorbed, Rockland, Item No. 600-401-379
Mouse Osteopontin/OPN Antibody, R&D Systems, Catalog#: AF808
Mouse anti-NeuN clone A60, Sigma Aldrich, MAB377
Donkey Anti-Chicken, Alexa 488 Conjugated, Jackson Immuno 703-545-155
Donkey anti-Mouse IgG (H+L) Highly Cross-Adsorbed Secondary Antibody, Alexa Fluor Plus 405, Thermo Fisher, A48257
Donkey anti-Rabbit IgG (H+L) Highly Cross-Adsorbed Secondary Antibody, Alexa Fluor Plus 594, Thermo Fisher, A32754
Donkey anti-Goat IgG (H+L) Cross-Adsorbed Secondary Antibody, Alexa Fluor 647, Thermo Fisher, A-21447
Abcam ab34771, Rabbit-anti-RFP
Millipore AP144P, Goat-anti-ChAT
Sigma A2052, Rabbit-anti-GABA

Validation

Links to datasheets from manufacturers detailing antibody validation are listed below:

AVES: https://cdn.shopify.com/s/files/1/0512/5793/4009/files/GFP_datasheet.pdf

Rockland: https://www.rockland.com/datasheet/?code=600-401-379

R&D Systems: https://resources.rndsystems.com/pdfs/datasheets/af808.pdf?v=20230413&_ga=2.75829141.1486737172.1681410426-2 76112423.1661781903

Sigma Aldrich: https://www.sigmaaldrich.com/US/en/product/mm/mab377

Jackson Immuno: https://www.jacksonimmuno.com/catalog/products/703-545-155

Donkey anti-Mouse: https://www.thermofisher.com/order/genome-database/dataSheetPdf?producttype=antibody&productsubtype=antibody_secondary&productId=A48257&version=288

Donkey anti-Rabbit: https://www.thermofisher.com/order/genome-database/dataSheetPdf?producttype=antibody&productsubtype=antibody_secondary&productId=A32754&version=288

Donkey anti-Goat: https://www.thermofisher.com/order/genome-database/dataSheetPdf?producttype=antibody&productsubtype=antibody_secondary&productId=A-2144 7&version=288

Abcam Rabbit-anti-RFP: https://www.abcam.com/products/primary-antibodies/biotin-rfp-antibody-ab34771.pdf

Millipore Goat-anti-ChAT: https://www.emdmillipore.com/US/en/product/Anti-Choline-Acetyltransferase-Antibody,MM_NF-AB144P

Sigma Rabbit-anti-GABA: https://www.sigmaaldrich.com/deepweb/assets/sigmaaldrich/product/documents/290/055/a2052dat.pdf

Animals and other research organisms

Policy information about studies involving animals; ARRIVE guidelines recommended for reporting animal research, and Sex and Gender in Research

Laboratory animals

Mice were provided food and water ad libitum, housed on a 12-hour light-dark schedule (7am-7pm light period) with no more than 5 mice of the same sex per cage, and allowed to acclimate for 1 week after arrival. Ambient temperature and humidity of housing facility: 71ºF +/- 3ºF; 35% - 70% +/- 5%

For sequencing experiments, 6 week old C57BL/6J mice were ordered from Jax, and housed in BCH's animal facility. For histology, 6 to 9 week old C57BL/6J and other Cre line (detailed in Methods section) were used for injection and sacrificed two to four weeks later

Wild animals	No wild animals were used in this study.
Reporting on sex	Nuclei suspensions were generated from 15 adult mice (8 female and 7 male) for 10x and 15 mice (6 female and 9 male) for SSv4. Sex based analyses were not conducted.
Field-collected samples	No field collected samples were used in this study.<br><br>Nuclei suspensions were generated from 15 adult mice (8 female and 7 male) for 10x and 15 mice (6 female and 9 male) for SSv4.
Ethics oversight	All experimental procedures were performed in compliance with animal protocols approved by the Institutional Animal Care and Use Committee at Boston Children's Hospital (Protocol #20-05-4165R).

Note that full information on the approval of the study protocol must also be provided in the manuscript.

Plants

Seed stocks	N/A
Novel plant genotypes	N/A
Authentication	N/A

Flow Cytometry

Plots

Confirm that:

☒ The axis labels state the marker and fluorochrome used (e.g. CD4-FITC).

☒ The axis scales are clearly visible. Include numbers along axes only for bottom left plot of group (a 'group' is an analysis of identical markers).

☒ All plots are contour plots with outliers or pseudocolor plots.

☒ A numerical value for number of cells or percentage (with statistics) is provided.

Methodology

Sample preparation	Generation of nuclei suspensions is described in detail in the Methods.
Instrument	For 10x, single SPN nuclei were sorted using a BD FACSARIA II with a 70 µm custom pressure nozzle (5O psi). For SSv4, single nuclei were sorted using a Sony SH800 Cell Sorter or MA900 Multi-Application Cell Sorter using a 100 µm chip.
Software	Sony Cell Sorter Software was used for the Sony SH800 and MA900 Cell sorters. BD FACSDiva Software was used for the BD FACSARIA II.
Cell population abundance	For a representative sample dissected from M1M2S1 cortex, GFP+ nuclei comprised 0.15% of all detected events, and GFP and/or mScarlet+ nuclei comprised 0.21% of all detected events, as shown in Extended Data Figure 3.
Gating strategy	We set FACS gating on forward scatter area - side scatter area plot (gate 1), forward scatter height - forward scatter area (gate 2), side scatter height - side scatter width (gate 3), DAPI area - forward scatter area (gate 4) and on fluorescent channels to include only GFP+ or mScarlet+ nuclei (gate 4), as shown in Extended Data Figure 3.

☒ Tick this box to confirm that a figure exemplifying the gating strategy is provided in the Supplementary Information.
