# Supplementary material for: A transcriptomic taxonomy of mouse brain-wide spinal projecting neurons
Source: Nature. 2023 Dec 13;624(7991):403–14. doi: 10.1038/s41586-023-06817-8 (PMC10719099; doi:10.1038/s41586-023-06817-8)
Supplement: Supplementary file 3 — Supplementary Tables 1–15 and table guide. [file 41586_2023_6817_MOESM3_ESM.zip › SI Table Guide.pdf]

Supplementary Table Legends

Supplementary Table 1: snRNAseq donor information. Mouse donors used in this study are listed alongside metadata, including the platform nuclei were profiled with, donor ID, strain, sex, and age. Multiple regions could be dissected per donor and are listed as ROI 1-8. GRN = gigantocellular reticular nucleus, PRN = pontine reticular nucleus, DPT = dorsal pontine tegmentum, L5CTX = cortical layer 5, RN = red nucleus, RFA = rostral forelimb area, M1 = primary motor cortex, M2 = secondary motor cortex, S1 = primary somatosensory cortex, S2 = secondary somatosensory cortex, HY = hypothalamus, MB = midbrain, CB = cerebellum, MED = medulla.

Supplementary Table 2: Marker genes of the 76 spinal projecting neuron types. Seurat's FindAllMarkers (MAST test) was used to find the marker genes of the 76 SPN types (10x and SSv4 data, N = 65,002). We tested only genes that were detected in a minimum of 25% of the nuclei (min.pct = 0.25) and that showed at least a 0.25-fold log-scale difference (logfc.threshold = 0.25) between the nuclei in the cluster and all other nuclei. Genes are annotated as 'significant' if the FDR adjusted p-value is less than 0.05.

Supplementary Table 3: Results of mapping the SPN snRNAseq taxonomy to the AIBS whole brain scRNAseq taxonomy - Subclass. The SPN taxonomy was mapped to the AIBS whole brain scRNAseq taxonomy. Here is SPN 'type' mapping to AIBS 'Subclass' level. Freq = frequency of mapped nuclei. Mappings with frequency of less than 3 were removed.

Supplementary Table 4: **Results of mapping the SPN snRNAseq taxonomy to the AIBS whole brain scRNAseq taxonomy - Supertype.** The SPN taxonomy was mapped to the AIBS whole brain scRNAseq taxonomy. Here is SPN 'type' mapping to AIBS 'Supertype' level. Freq = frequency of mapped nuclei. Mappings with frequency of less than 3 were removed.

Supplementary Table 5: Results of mapping the SPN snRNAseq taxonomy to the AIBS whole brain scRNAseq taxonomy - Cluster. The SPN taxonomy was mapped to the AIBS whole brain scRNAseq taxonomy. Here is SPN 'type' mapping to AIBS 'Cluster' level. Freq = frequency of mapped nuclei. Mappings with frequency of less than 3 were removed.

Supplementary Table 6: Differentially expressed genes among corticospinal neurons arising from M1M2S2, RFA, and S2. Seurat's FindAllMarkers (MAST test) was used to find differentially expressed genes among corticospinal neurons arising from M1M2S1, RFA, and S2 areas of layer 5 cortex (10x data, N = 28,740). We tested only genes that were detected in a minimum of 25% of the nuclei (min.pct = 0.25) and that showed at least a 0.25-fold log-scale difference (logfc.threshold = 0.25) between the nuclei in the cluster and all other nuclei. Genes are annotated as 'significant' if the FDR adjusted p-value is less than 0.05.

Supplementary Table 7: Marker genes of the pontomedullary spinal projecting neuron types. Seurat's FindAllMarkers (MAST test) was used to find the marker genes of the 53 pontomedullary SPN types (10x dataset, N = 22,100). We tested only genes that were detected in a minimum of 10% of the nuclei (min.pct = 0.10) and that showed at least a 0.25-fold log-scale

difference (logfc.threshold = 0.25) between the nuclei in the cluster and all other nuclei. Genes are annotated as ‘significant’ if the FDR adjusted p-value is less than 0.05.

Supplementary Table 8: Differentially expressed genes between all cervical- vs. dual- and lumbar- spinal projecting neurons. Seurat’s FindMarkers (MAST test) was used to find differentially expressed genes between cervical vs. dual and lumbar projecting SPNs in each ROI containing both projection types (i.e., M1M2S1, HY, MB, CB, PONS, MED). We tested only genes that were detected in a minimum of 10% of the nuclei (min.pct = 0.10) and that showed at least a 0.05-fold log-scale difference (logfc.threshold = 0.05) between the two groups. Loose $\log_2$(Fold Change) criteria was used to capture more differentially expressed genes across SPN ROI and to account for relatively low sequencing depth 10x data. We then removed any genes that were not significant (FDR adjusted p-value greater than 0.05) and found the intersection across the 6 lists, to yield a final list of genes that are significantly differentially expressed in each ROI. Positive $\log_2$(Fold Change) indicates differentially expressed in cervical-projecting neurons.

Supplementary Table 9: Differentially expressed genes between cervical- vs. dual- and lumbar- projecting corticospinal neurons. Seurat’s FindMarkers (MAST test) was used to find differentially expressed genes between cervical vs. dual/lumbar projecting corticospinal neurons (SSv4 data, N = 1,009 nuclei). We tested only genes that were detected in a minimum of 10% of the nuclei (min.pct = 0.10) and that showed at least a 0.25-fold log-scale difference (logfc.threshold = 0.25) between the two groups. Genes are annotated as ‘significant’ if the FDR adjusted p-value is less than 0.05. Positive $\log_2$(Fold Change) indicates differentially expressed in cervical-projecting neurons.

Supplementary Table 10: Differentially expressed genes between cervical- vs. dual- and lumbar- projecting rubrospinal neurons. Seurat’s FindMarkers (MAST test) was used to find differentially expressed genes between cervical vs. dual/lumbar projecting rubrospinal neurons (SSv4 data, N = 1,031 nuclei). We tested only genes that were detected in a minimum of 10% of the nuclei (min.pct = 0.10) and that showed at least a 0.25-fold log-scale difference (logfc.threshold = 0.25) between the two groups. Genes are annotated as ‘significant’ if the FDR adjusted p-value is less than 0.05. Positive $\log_2$(Fold Change) indicates differentially expressed in cervical-projecting neurons.

Supplementary Table 11: Differentially expressed genes between lumbar- and dual-projecting rubrospinal neurons. Seurat’s FindMarkers (MAST test) was used to find differentially expressed genes between lumbar vs. dual projecting rubrospinal neurons (SSv4 data, N = 1,031 nuclei). We tested only genes that were detected in a minimum of 10% of the nuclei (min.pct = 0.10) and that showed at least a 0.25-fold log-scale difference (logfc.threshold = 0.25) between the two groups. Genes are annotated as ‘significant’ if the FDR adjusted p-value is less than 0.05. Positive $\log_2$(Fold Change) indicates differentially expressed in lumbar-projecting neurons.

Supplementary Table 12: Differentially expressed genes between Spp1 positive and negative rubrospinal neurons. Seurat’s FindAllMarkers (MAST test) was used to find

differentially expressed genes between Spp1 positive and negative rubrospinal neurons (SSv4 data, N = 1,031 nuclei). We tested only genes that were detected in a minimum of 10% of the nuclei (min.pct = 0.10) and that showed at least a 0.25-fold log-scale difference (logfc.threshold = 0.25) between the two groups. Genes are annotated as 'significant' if the FDR adjusted p-value is less than 0.05.

Supplementary Table 13: Summary of electrophysiological data. Summary of number of cells measured and statistical tests for data appearing in Figure 6 and Extended Data Figure 16.

Supplementary Table 14: Mouse lines used for Cre-dependent retrograde labeling. Summary of mouse lines used for retrograde labeling with AAVs expressing Cre-dependent GFP. The Jackson Lab Strain and MGI number is provided for publicly available strains, and name of donating lab is listed for non-publicly available strains.

Supplementary Table 15: Estimated multiplet rate of 10x snRNAseq samples. The multiplet rate (%) was estimated for each 10x snRNAseq sample. From the reported number of nuclei sorted from the FACS machine, estimated sort recovery was calculated based on an estimated 70% recovery rate. Multiplet rate was then calculated from the Multiplet Rate Table provided by 10x Genomics. It is important to note these rates are not exact calculations, as both sort recovery and multiplet rate are *estimations*.
